# Identification and Characterization of Circular RNAs (circRNAs) Using RNA-Seq in Two Breeds of Cashmere Goats

**DOI:** 10.3390/genes14020331

**Published:** 2023-01-27

**Authors:** Liyan Hu, Jiqing Wang, Yuzhu Luo, Xiu Liu, Shaobin Li, Zhiyun Hao, Fangfang Zhao, Mingna Li, Bingang Shi, Yuanhua Gu

**Affiliations:** Gansu Key Laboratory of Herbivorous Animal Biotechnology, College of Animal Science and Technology, Gansu Agricultural University, Lanzhou 730070, China

**Keywords:** circular RNA (circRNA), cashmere fiber, cashmere goats, RNA-seq, skin tissue

## Abstract

Circular RNA (circRNA) is a type of non-coding RNA generated from back-splicing the reactions of linear RNA. It plays an important role in various cellular and biological processes. However, there are few studies about the regulatory effect of circRNAs on cashmere fiber traits in cashmere goats. In this study, the expression profiles of circRNAs in skin tissue were compared between Liaoning cashmere (LC) goats and Ziwuling black (ZB) goats, with a significant difference in cashmere fiber yield, cashmere fiber diameter, and cashmere fiber color, using RNA-seq. A total of 11,613 circRNAs were expressed in the caprine skin tissue, and their type, chromosomal distribution, and length distribution were characterized. A total of 115 up-regulated circRNAs and 146 down-regulated circRNAs in LC goats were screened compared to ZB goats. The authenticity of 10 differentially expressed circRNAs was validated by detecting their expression levels and the head-to-tail splice junction using RT-PCR and DNA sequencing, respectively. The parent genes of differentially expressed circRNA were mainly enriched in some Gene Ontology (GO) terms and pathways related to cashmere fiber traits, such as the canonical Wnt signaling pathway, which is involved in the regulation of cell promotion, stem cell proliferation, Wnt signaling pathway regulation, epithelial morphogenesis, MAPK signaling pathway, and cell adhesion molecules pathway. Eight differentially expressed circRNAs were further selected to construct a circRNA-miRNA network, and some miRNAs that were previously reported as related to fiber traits were found in the network. This study provides a deep understanding of the roles of circRNAs in the regulation of cashmere fiber traits in cashmere goats and the involvement of differential splicing in phenotypic expression according to breed and region.

## 1. Introduction

Circular RNA (circRNA) is a novel type of non-coding RNA that has attracted extensive attention in recent years. As there is a covalent closed-loop structure, circRNA is more stable and conservative than its parent linear RNA [1]. It has been confirmed that most circRNAs mainly act as microRNA (miRNA) sponges to relieve the inhibition of the target mRNAs in expression derived from miRNAs [2]. These circRNAs also play a biological function in increasing the expression levels of the target genes. Alternatively, some circRNAs can interact with RNA-binding proteins to regulate the expression of their parent genes by regulating the transcription of RNA polymerase II or modifying the methylation of N6-methyladenosine (m^6^A) [3,4]. Additionally, a small number of circRNAs have the ability to translate into functional proteins [5]. In this context, it can be reasonably suggested that circRNAs can become powerful tools for accurately elucidating the biological mechanisms underlying complex economic traits in domestic animals.

Skin is one of the largest organs and can produce wool or cashmere fibers in sheep or goats. In cashmere goats, the fleece is composed of cashmere and larger-fiber-diameter “guard hairs”, which are produced by the secondary fiber follicles and the primary fiber follicles, respectively. It is well known that the structure of the skin tissue and hair follicle development determine the yield and quality of cashmere fibers. Many studies have found that circRNAs play roles in a variety of biological processes in organisms, including the regulation of the growth and development of skin and hair follicles in cashmere goats. For example, circRNA-1967, circRNA-1926, and circRNA-0100 exhibited significantly higher expression at anagen than at telogen in secondary hair follicles of cashmere goats. Moreover, the three circRNAs regulated the expression levels of *LEF1*, *CDK19*, and *KLF5* by competitively binding miR-93-3p, miR-148a/b-3p, and miR-153-3p, respectively, eventually promoting the differentiation of secondary hair follicle stem cells into a hair follicle lineage in cashmere goats [6,7,8].

Additionally, in recent years, RNA-seq has been widely used to construct expression profiles of circRNAs and investigate their roles in the skin tissue of cashmere goats and sheep. For example, a total of 32 differentially expressed circRNAs were identified in skin tissue between two cashmere goat breeds with divergent cashmere fiber traits [9]. Similarly, another study found 32 differentially expressed circRNAs in the skin tissue between Liaoning cashmere goats and Inner Mongolia cashmere goats, and their parent genes were significantly enriched in keratinization, intermediate filament organization, spindle midzone, Wnt-protein binding, Wnt-activated receptor activity, and the negative regulation of stress fiber assembly, which were related to the formation and growth of cashmere fiber [10].

Liaoning cashmere (LC) goats and Ziwuling black (ZB) goats are famous dual-purpose breeds for cashmere fiber and meat in China, and the two breeds are also of economic importance in the regions in which they are raised. There are significant differences in cashmere production performance and quality between LC goats and ZB goats. Namely, the cashmere fiber diameter of ZB goats is smaller than that of LC goats, but LC goats have a higher cashmere yield than ZB goats. Meanwhile, LC goats produce white cashmere fibers, while ZB goats produce purple cashmere fibers. However, the biological mechanism that regulates the differences remains unclear. Accordingly, in this study, RNA-seq was used to profile the circRNA expression of caprine skin tissue. The differentially expressed circRNAs were also identified between LC goats and ZB goats, and their parent genes were used to perform a functional enrichment analysis. A circRNA-miRNA was constructed to screen for functional circRNAs acting as miRNA sponges. The results will uncover the molecular mechanisms underlying the differences in cashmere fiber traits between LC and ZB goats and reveal potential functions of circRNAs in the regulation of cashmere fiber traits in goats.

## 2. Materials and Methods

### 2.1. Ethics Statement

All animal experiments involved in this study were approved by the Animal Experimental Ethics Committee of Gansu Agricultural University with the approval number of GSAU-ETH-AST-2021-028. 

### 2.2. Sample Collection

At the Yunfeng Cashmere Goat Breeding Company (Huan county, Qingyang, China), six healthy, three-year-old male LC goats and six healthy, three-year-old male ZB goats were selected for investigation. All goats were used for cashmere fiber production and raised under the same feeding management level. The cashmere fiber weight and cashmere fiber diameter of the LC goats selected in the study were 1539 ± 26.6 g and 15.9 ± 0.07 μm, while the ZB goats were 395 ± 17.5 g and 13.9 ± 0.03 μm, respectively.

In mid-August, when these goats were in the anagen phase of cashmere fiber growth, the skin sample for each goat was collected using a surgical biopsy. Briefly, the wool on the surface of the skin was shaved and then wiped with 75% alcohol. After local anesthesia, about 3 cm^2^ of skin on the right side of each goat was grasped with sterile pliers and quickly cut off with a sterile scalpel. The goats were immediately treated with Yunnan Baiyao (Yunnan Baiyao Group Co., Ltd., Kunming, China) to stop the bleeding. The skin samples collected were washed with physiological saline and RNase water and then cut into small pieces measuring 1 cm × 2 cm. The samples were quickly frozen in liquid nitrogen.

### 2.3. RNA Extraction and Sequencing

Total RNA was isolated and purified from twelve caprine skin tissue samples using Trizol reagent (Invitrogen, Carlsbad, CA, USA). The purity and concentration of the RNA extracted were checked using a Nanodrop 2000 (Thermo Scientific, Waltham, MA, USA) and a Qubit^®^ 2.0 fluorometer (Life Technologies, Carlsbad, CA, USA), respectively. The RNA integrity number (RIN) measured by an Agilent 2100 Bioanalyzer (Agilent, Palo Alto, CA, USA) was used to screen high-quality RNA samples (RIN > 7). Ribosomal RNA (rRNA) was depleted from these high-quality RNA samples using a Ribo-Zero Gold rRNA Removal Kit (Illumina, San Diego, CA, USA), and the remaining RNA was used to produce complementary DNA (cDNA) libraries using a NEBNext Ultra RNA Library Prep Kit (New England Biolabs, Ipswich, MA, USA). The cDNA libraries constructed were paired-end sequenced using an Illumina HiSeq2500 sequencer (Illumina, San Diego, CA, USA) at the Gene *Denovo* Biotechnology Co., Ltd. (Guangzhou, China).

### 2.4. RNA-Seq Data Analysis

For raw reads obtained in the FASTQ format, their unqualified reads were removed to obtain clean reads, including adapter reads, reads containing poly (A) bases, low-quality reads with quality scores < Q20, and reads containing more than 10% of unknown nucleotides. The clean reads were mapped against Caprine Genome Assembly ARS1.2 (https://www.ncbi.nlm.nih.gov/assembly/GCF_001704415.2, accessed on 27 June 2020) using HISAT2 v2.1.0. For the reads that were unmapped against Caprine Genome Assembly ARS1, 20 bp from both ends were intercepted as anchor reads. The anchor reads were aligned to the reference genome again using bowtie2 v2.2.8 and the alignment results were used to identify circRNAs using Find_circ [11]. The characteristics of the circRNAs identified were analyzed by counting their length, type, and chromosome distribution.

The expression levels of the annotated circRNAs were normalized with the Reads Per Million mapped reads (RPM). The parameters of fold change > 2.0 and *p* < 0.05 were set as the threshold to screen differentially expressed circRNAs in skin tissue between LC goats and ZB goats using the DESeq v2.0.

### 2.5. Validation of the Authenticity of circRNAs Using RT-PCR and Sanger Sequencing

The authenticity of the circRNAs annotated in the study was verified using reverse transcriptase-PCR (RT-PCR) and DNA sequencing. Briefly, a total of 10 differentially expressed circRNAs were selected. The 12 RNA samples that were used for RNA-seq were also used to produce cDNA using an Evo M-MLV RT mix kit (Accurate Biology, Changsha, Hunan, China). In addition, the cDNA was amplified using divergent primers (Table 1). The RT-PCR products were detected by 1.5% agarose gel electrophoresis and then sequenced using Sanger sequencing. To validate the presence of the head-to-tail splice junction of circRNAs, the sequences obtained from Sanger sequencing were aligned to sequences from RNA-seq and Caprine Genome Assembly ARS1.2 using MEGA 5.0.

### 2.6. Validation of the Reliability of RNA-Seq by RT-qPCR

To confirm the reliability of the RNA-seq results, the same 10 differentially expressed circRNAs that were analyzed using RT-PCR were selected to perform RT-quantitative PCR (RT-qPCR). The RNA samples originally extracted for the RNA-seq analysis were used to produce cDNA using an Evo M-MLV RT mix kit. The RT-qPCR was performed in triplicate using SYBR^®^ Green PCR Master Mix (Takara, Dalian, China) on an Applied Biosystems QuantStudio^®^ 6 Flex (Thermo Lifetech, Waltham, MA, USA). Caprine *GAPDH* was selected as an internal reference gene to normalize the expression levels of these circRNAs [12]. Their relative expression levels were calculated using the 2^−ΔΔCt^ method. 

### 2.7. GO and KEGG Enrichment Analysis of the Parent Genes of Differentially Expressed circRNAs

The functional enrichment of the parent genes of differentially expressed circRNAs was analyzed using the Gene Ontology database (http://www.geneontology.org/, accessed on 7 November 2020) and the Kyoto Encyclopedia of Genes and Genomes database (http://www.kegg.jp/kegg, accessed on 7 November 2020). The GO terms and KEGG pathways with *p* < 0.05 were considered significantly enriched based on a hypergeometric test.

### 2.8. Construction of circRNA-miRNA Regulatory Network

Eight differentially expressed circRNAs belonging to the annot_exons type were selected, and Mireap v0.2 (https://sourceforge.net/projects/mireap/, accessed on 23 May 2022), Miranda v3.3a, (http://www.microrna.org/microrna/home.do, accessed on 23 May, 2022) and targetscan v3.1 (http://www.targetscan.org/, accessed on 23 May, 2022) were used to predict the binding sites of the target miRNAs of the circRNAs. The predicted results overlapped. Of all the miRNAs predicted, the miRNAs related to hair follicle growth and development were chosen. A circRNA-miRNA regulatory network was constructed using starBase v3.0 and then visualized using Cytoscape v3.5.1.

## 3. Results

### 3.1. Identification and Characterization of circRNAs in Skin Tissue of Cashmere Goats

**Figure 1:**
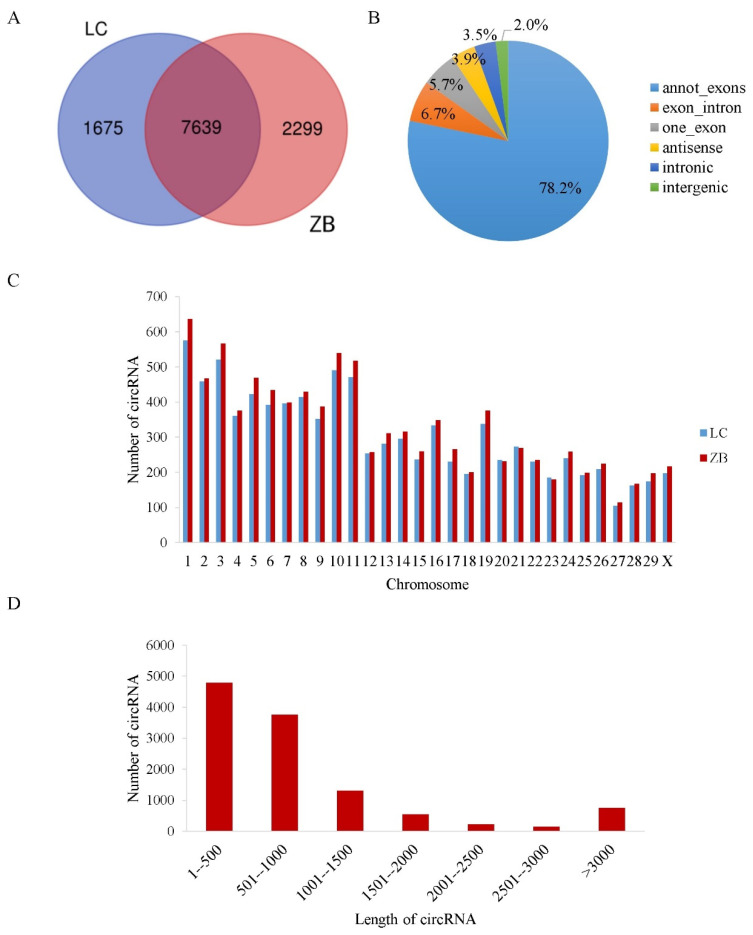
General characteristics of circRNAs in the skin tissue of Liaoning cashmere (LC) goats and Ziwuling black (ZB) goats. (**A**) A Venn diagram showing the number of expressed circRNAs in the caprine skin tissue of LC goats and ZB goats. (**B**) The types of circRNAs in the caprine skin tissue. (**C**) The chromosomal distribution of circRNAs identified in the caprine skin tissue. (**D**) The length distribution of circRNAs in the caprine skin tissue.

The raw reads obtained in this study have been submitted to the GenBank database with accession numbers SRR19879981-SRR19879992. After removing unqualified reads, a total of 84,025,004 and 92,946,813 clean reads were obtained in skin tissue collected from LC and ZB goats, respectively, of which 84,011,916 and 92,921,492 reads mapped well to the Caprine Genome Assembly, with a unique mapping rate of 93.46% and 92.41%, respectively. A total of 11,613 circRNAs were detected from the caprine skin tissue, with 7639 circRNAs being co-expressed in both breeds (Figure 1A). In other words, there were 9314 and 9938 circRNAs identified in the skin tissue of LC and ZB goats, respectively.

Of the six types of circRNAs classified, annot_exons were the most common sequences, accounting for 78.2% of the total circRNAs identified. This was followed by exon_intron, one_exon, and antisense, with an average proportion of 6.7%, 5.7%, and 3.9%, respectively. Intronic and intergenic sequences were the least common types, with an average proportion of 3.5% and 2.0%, respectively (Figure 1B). The circRNAs found in the caprine skin tissues were distributed across all the caprine chromosomes, including the X chromosome. The most circRNAs were distributed on chromosomes 1, 3, 10, and 11, while the least circRNAs were distributed on chromosome 27 (Figure 1C). The majority of the circRNAs had a length of less than 1000 bp (Figure 1D).

### 3.2. Validation of the Authenticity of circRNAs Identified in the Caprine Skin Tissue

**Figure 2:**
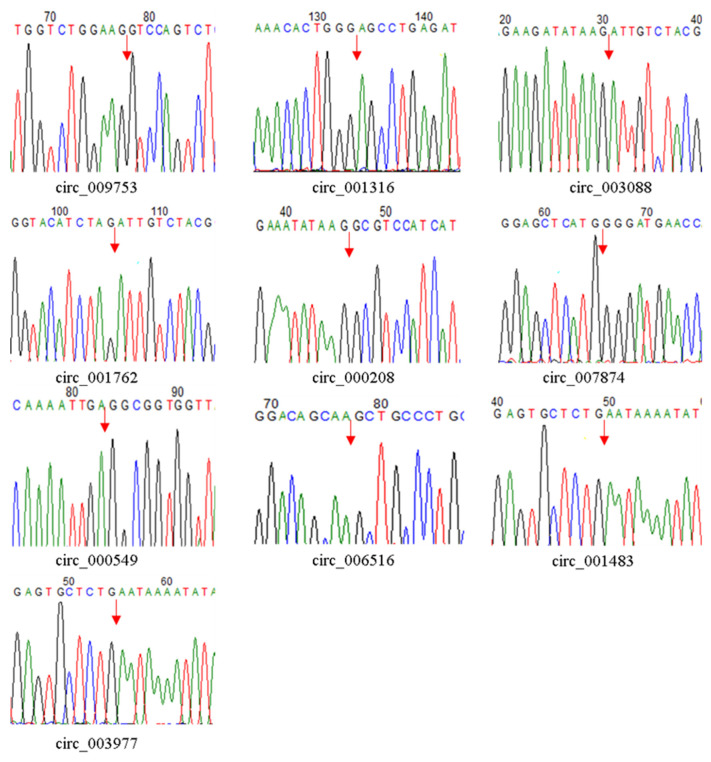
Confirmation of the back-splicing junctions for 10 circRNAs selected using Sanger sequencing. A red arrow represents the junction site on the DNA sequence chromatograms.

RT-PCR and Sanger sequencing were used to verify the authenticity of the circRNAs identified using an RNA-Seq analysis. The RT-PCR results found that all 10 circRNAs could be expressed, with an expected size band on agarose gel electrophoresis. The DNA sequencing results verified the presence of the head-to-tail splice junction sequences as suggested by RNA-Seq (Figure 2).

### 3.3. Analysis and Validation of Differentially Expressed circRNAs between the Two Caprine Breeds

Of all the 11,613 circRNAs identified in caprine skin tissue, 261 circRNAs were found to be differentially expressed between LC and ZB goats, including 115 up-regulated circRNAs and 146 down-regulated circRNAs in the skin tissue of LC goats when compared to ZB goats (Supplementary File S1). The five most up-regulated circRNAs in LC goats were circ_003977, circ_001161, circ_004733, circ_003577, and circ_000549, while the five most down-regulated circRNAs in LC goats were circ_007344, circ_001762, circ_003528, circ_000831, and circ_008802. Notably, of the differentially expressed circRNAs detected, 50 circRNAs were only expressed in ZB goats, while 29 circRNAs were only expressed in LC goats.

**Figure 3:**
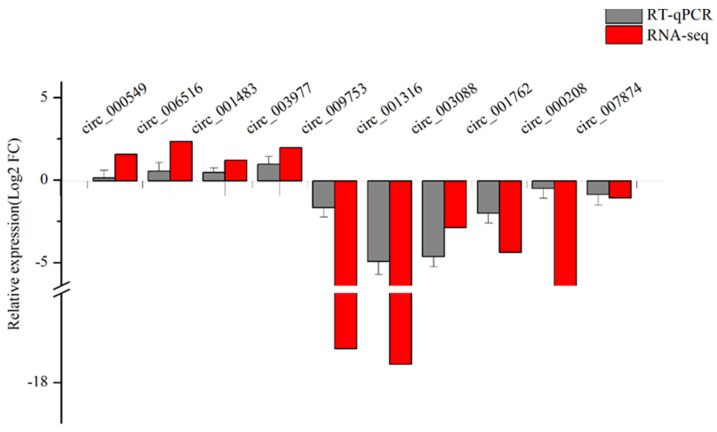
The comparisons of the expression levels of 10 differentially expressed circRNAs obtained from RNA–seq with those obtained from RT–qPCR analysis.

To further validate the reliability of the RNA-seq data, the 10 differentially expressed circRNAs that were used to verify the authenticity described above were selected for RT-qPCR analysis. As shown in Figure 3, the relative expression levels of circ_000549, circ_006516, circ_001483, and circ_003977 in LC goats were higher than those in ZB goat skin tissue. However, the relative expression levels of circ_009753, circ_001316, circ_003088, circ_001762, circ_000208, and circ_007874 in the skin tissue of LC goats were lower than those in ZB goats. The results indicate that the RT-qPCR results of these circRNAs in caprine skin tissue were consistent with those obtained from RNA-seq, which further illustrated the reliability of the RNA-seq data (Figure 3).

### 3.4. Function Enrichment Analysis of the Parent Genes of Differentially Expressed circRNAs

**Figure 4:**
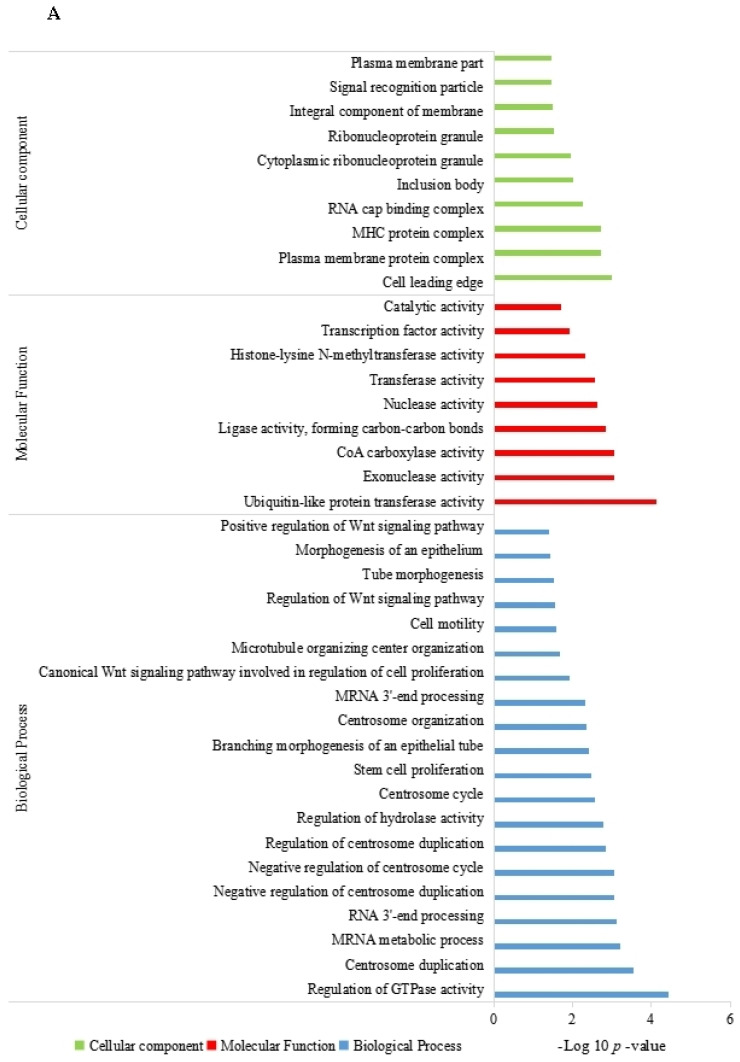

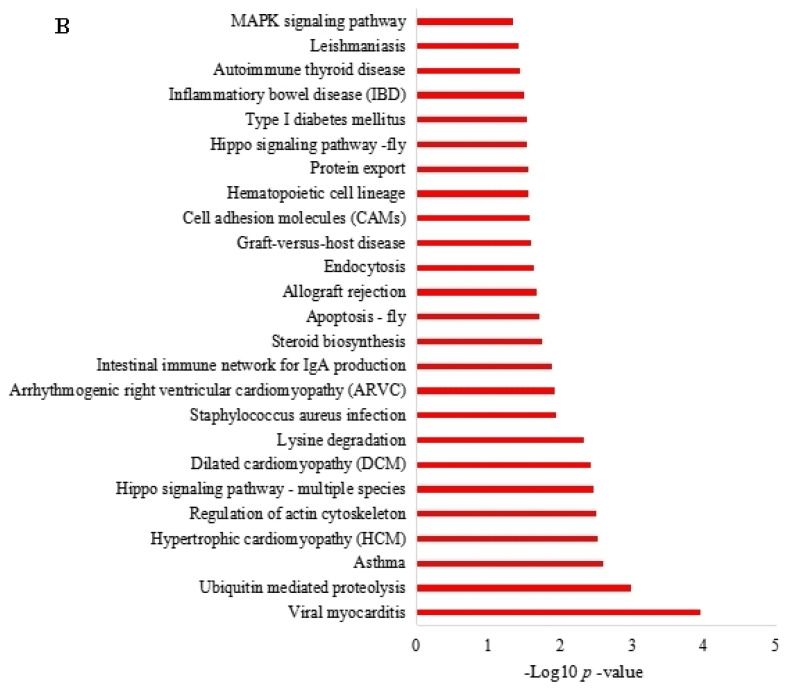
Gene ontology (**A**) and KEGG enrichment (**B**) analysis of the parent genes of differentially expressed circRNAs.

In order to further explore how these differentially expressed circRNAs regulate differences in cashmere fiber traits between LC goats and ZB goats, GO and KEGG analyses were carried out for their parent genes. A total of 240 parent genes were annotated for the 261 differentially expressed circRNAs. The most significant GO term with the lowest *p* value was regulation of GTPase activity (*p* = 3.74 × 10^−5^), followed by ubiquitin-like protein transferase activity (*p* = 7.51 × 10^−5^), centrosome duplication (*p* = 0.0003), and mRNA metabolic process (*p* = 0.0006). It was notable that several important GO terms related to hair follicle growth were also found, including the canonical Wnt signaling pathway involved in the regulation of cell promotion (*p* = 0.0124), stem cell proliferation (*p* = 0.0033), Wnt signaling pathway regulation (*p* = 0.0275), and epithelial morphogenesis (*p* = 0.0370) (Figure 4A).

The parent genes of the differentially expressed circRNAs were significantly enriched in 25 KEGG pathways. The most significant pathway with the lowest *p* value was viral myocarditis (*p* = 0.0001), followed by ubiquitin-mediated proteolysis (*p* = 0.0011), asthma (*p* = 0.0025), and hypertrophic cardiomyopathy (*p* = 0.0031) (Figure 4B). Interestingly, the MAPK signaling pathway and the cell adhesion molecules pathway associated with fiber traits were also enriched.

### 3.5. The miRNA Sponges Analysis of Differentially Expressed circRNAs

**Figure 5:**
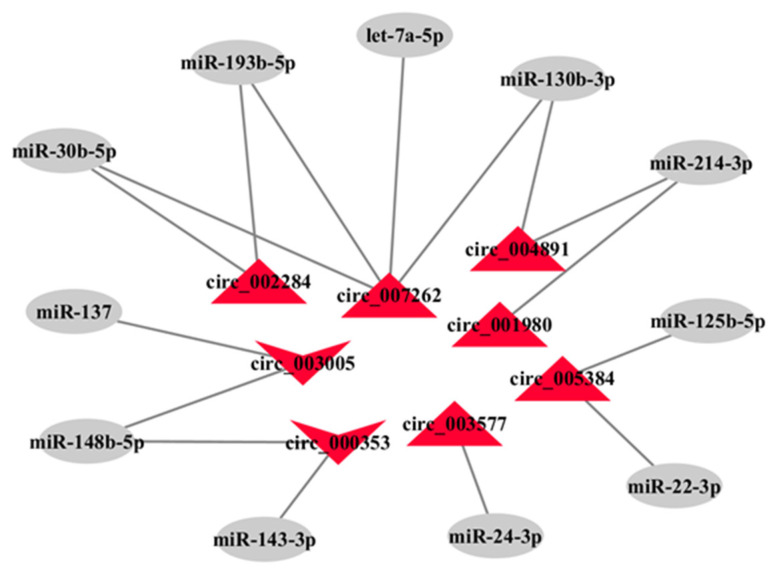
The interaction network of circRNA-miRNA. The red triangles and inverted triangles indicate up-regulated and down-regulated circRNAs in the skin tissues of Liaoning cashmere (LC) goats compared to Ziwuling black (ZB) goats, respectively. The gray circles represent predicted target miRNAs for circRNA.

It is well known that annot_exons circRNAs located in the cytoplasm can function as miRNA sponges. To investigate potential miRNA sponge roles of circRNAs in caprine skin tissue, 8 differentially expressed annot_exons circRNAs were selected, including 6 up-regulated circRNAs (circ_003577, circ_001980, circ_007262, circ_005384, circ_004891, and circ_002284) and 2 down-regulated circRNAs (circ_003005 and circ_000353) in LC goats. Among them, circ_003577, circ_007262, and circ_001980 were expressed only in LC goats, while circ_000353 was expressed only in ZB goats. The 8 circRNAs have a total of 137 miRNA binding sites. Based on the roles of the miRNAs in hair follicle growth and development reported previously [4,13,14], 11 sponge miRNAs were further selected to construct a circRNA-miRNA network (Figure 5). As shown in Figure 5, 16 pairs of circRNA-miRNAs were screened.

## 4. Discussion

As a high-quality textile material, cashmere fiber is produced from the secondary hair follicles of skin tissue in cashmere goats [15]. The secondary hair follicle development involves dynamic and cyclical periods of anagen, catagen, and telogen, and cashmere fibers therefore also show seasonal growth and shedding [16]. Previous studies have shown that the periodic activities of hair follicles regulate the growth of cashmere fiber. Either prolonging the anagen phase of secondary hair follicles or shortening their catagen and telogen phases can effectively increase cashmere fiber growth time, eventually leading to increases in cashmere yield [17,18]. In this context, it is of great significance to study the development of secondary hair follicles for the improvement of the yield and quality of cashmere fiber. Recent studies have found that non-coding RNAs have certain regulatory roles in hair follicle growth and development and cashmere fiber traits [19,20]. Therefore, the expression profile of circRNAs in skin tissue and their effect on cashmere traits and hair follicle development were investigated.

In this study, a total of 11,613 circRNAs were identified by RNA-seq in the skin tissue of LC and ZB goats during the anagen period. The number of circRNAs identified was similar to what was investigated by Zheng et al. [10], who found 13,320 circRNAs in the skin tissue of LC goats during the anagen period. However, the number of circRNAs expressed in the study was less than that reported in a previous study of skin tissue in Inner Mongolia cashmere goats at four different embryonic stages, with 21,784 circRNAs being identified [21]. This indicates breed-specific and time-specific expression patterns of circRNAs in goats, which is consistent with previous findings of circRNAs [22,23,24]. In this study, the differential expression of circRNAs also confirmed the breed-specific pattern, as 29 and 50 circRNAs were only expressed in the skin tissue of LC and ZB goats, respectively.

It was notable that 78.2% of the circRNAs identified were of the annot_exons type, which located in more than two exon regions of the coding genes. This indicates the preference of some protein-coding genes for loop formation [25]. This was also consistent with the results obtained from Inner Mongolian cashmere goats [21]. Meanwhile, caprine chromosome 1 produced the most circRNAs in this study. This was not surprising, as that chromosome is the largest in size when compared to other chromosomes in the goat genome. The study conducted by Shen et al. [24] in caprine *longissimus dorsi* muscle tissue also confirmed the result.

The up-regulated circ_006516 in LC goats attracted our attention as it had the highest expression level in LC goats. The circRNA is derived from *KRT33A*, also known as *KIFⅠ*. *KIFⅠ* is a member of the keratin intermediate filament family [26]. Keratin intermediate filament proteins are one of the main structural proteins of cashmere fibers and therefore determine the physico-mechanical characteristics of cashmere fiber [27]. As one of the down-regulated circRNAs in LC goats, circ_005116 is derived from *KRT84*. *KRT84* encodes the type II of hair keratin in the fibrous cuticles that are assembled in a highly organized fashion into keratin intermediate filaments [28,29]. Variations in genes encoding keratin have been reported to be associated with cashmere fiber diameter and curvature [30,31]. It was notable that of the differentially expressed circRNAs identified in this study, circ_001980 was only expressed in the skin tissue of LC goats, while circ_009498 was only expressed in ZB goats. The parent genes of circ_001980 and circ_009498 are *CDK19* and *FGFR2*, respectively. *CDK19* and *CDK8* have high amino acid conservation, so they are also called *CDK8L*. *CDK8* was required for Wnt/β-catenin signaling activation, which is important for hair follicle generation, differentiation of hair follicle stem cells, and hair stem cell growth [8,32]. It is possible that circ_001980 and circ_009498 originated from *CDK19*, and *FGFR2* may be involved in the regulation of the differences in cashmere fiber between LC and ZB goats. Taken together, it was therefore inferred that specifically expressed circ_001980 and circ_009498 in LC and ZB goats, respectively, may be responsible for the differences in cashmere fiber traits between LC goats and ZB goats.

In order to further explore the potential regulatory effect of circRNAs on cashmere fiber traits, GO and KEGG functional enrichment analyses were applied to their parent genes. Of the GO terms enriched by the parent genes, morphogenesis of an epithelium (GO:0002009), microtubule organizing center organization (GO:0031023), developmental growth involved in morphogenesis (GO:0060560), plasma membrane part (GO:0044459), regulation of Wnt signaling pathway (GO:0030111), and cell motility (GO:0048870) were involved in the development and formation of cashmere fibers. For example, Wnt signaling is the most critical pathway to induce hair follicle development [33]. Wnt signaling is also thought to be involved in the regulation of melatonin on hair follicle development. Melatonin has been found to promote cashmere fiber growth, eventually leading to increases in cashmere fiber yield [34,35,36]. Similarly, stem cell proliferation is closely related to the growth and development of hair follicles and the growth and formation of cashmere fiber. Proliferation and differentiation of hair follicle stem cells has been confirmed as an important condition for hair follicles to enter the growth stage and the main power source of hair regeneration [37,38]. Of the KEGG pathways enriched by the parent genes, the MAPK signaling pathway is an important pathway for the growth and development of mammalian hair follicles, as well as melanogenesis [39,40]. In addition, the parent genes of differentially expressed circRNAs identified in this study were also enriched in the cell adhesion molecule pathway. Cell adhesion molecules are proteins on the surface of cells that can bind to other cells or adhere to the extracellular matrix [41]. The extracellular matrix affects the quality of cashmere fiber by regulating the growth of hair follicles [42]. Therefore, it can be concluded that cell adhesion molecules also have certain effects on cashmere fiber quality. Taken together, the differentially expressed circRNAs found in the study may affect cashmere fiber traits by regulating the GO terms and KEGG pathways involved in their parent genes.

It has been confirmed that circRNA can regulate the expression level of the target genes by acting as a ‘sponge’ for miRNA. It was therefore important to investigate the interaction between specific circRNA and the sponge miRNA. In the study, some predicted target miRNAs of circRNAs have been reported to be involved in the regulation of cashmere fiber traits and hair follicle development. For example, as one of the most up-regulated circRNAs in LC goats, circ_003577 would target miR-24-3p (Figure 5). It has been found that miR-24-3p can change the structure of hair follicles in mice by inhibiting the expression of the hair keratinocyte stemness regulator *Tcf-3*, which causes a decreased cashmere fiber diameter [14]. This suggests that up-regulated circ_003577 may result in an increased cashmere fiber diameter of LC goats by decreasing the inhibited effect of miR-24-3p on cashmere fiber diameter. Furthermore, up-regulated circ_005384 in LC goats would target miR-22-3p and miR-125b-5p (Figure 5). The miR-22-3p targets *STK40* to inhibit MEF2-ALP activity, which is beneficial to hair follicle stem cell differentiation and hair growth [13]. Similarly, miR-125b-5p was found to inhibit cashmere fiber growth as it inhibited the regeneration and transformation in the fiber growth cycle by targeting *FGF5* and *TNF-α* [43]. It was speculated that up-regulated circ_005384 in LC goats may promote fiber growth, which leads to an increased cashmere fiber yield by sponging the inhibited effect of miR-125b-5p on cashmere fiber growth. Taken together, these results may explain why up-regulated circ_003577 and circ_005384 may produce a higher cashmere fiber yield and a larger diameter in LC goats.

It was notable that some miRNAs exhibited in the circRNA-miRNA interaction network were related to the cashmere fiber color. For example, circ_000353 was not only a down-regulated circRNA in LC goats but was also only expressed in the skin tissue of ZB goats. The circRNA would target miR-143-3p (Figure 5). It has been reported that miR-143-3p can reduce the production of melanin by suppressing the expression of TGF-β- activated kinase1 (*TAK1*) [44]. Down-regulated circ_000353 may therefore lead to white cashmere fiber in LC goats by lessening the inhibiting effect of miR-143-3p on melanosis. Similarly, miR-137 targeted by circ_003005 (Figure 5) inhibited the formation of melanin by negatively regulating the expression of *c-KIT* and *TYRP2* in the SCF/c-Kit signaling pathway, thus participating in the regulation of the hair color of mice [45]. Similar to circ_000353, down-regulated circ_003005 may result in white cashmere fiber from LC goats by lessening the inhibiting effect of miR-137 on the formation of melanin. Taken together, these suggest that the differential expression of the circRNAs described above may be responsible for the difference in cashmere fiber color between LC and ZB goats by acting as miRNA sponges.

## 5. Conclusions

**Figure 6:**
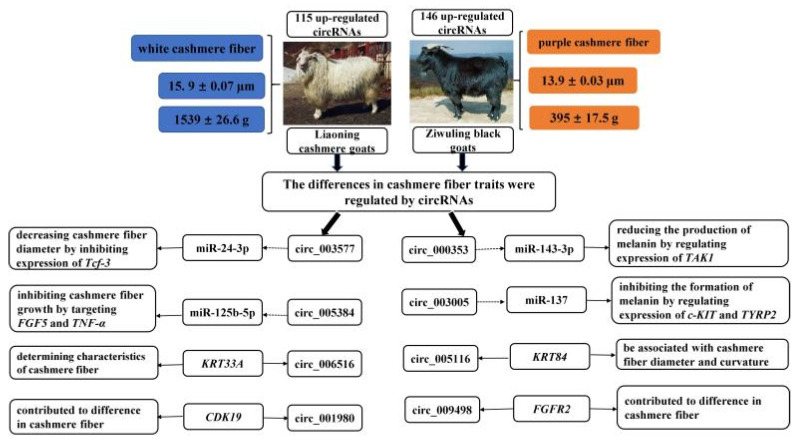
The regulatory mechanism of circRNAs on differences in cashmere fiber traits between LC and ZB goats.

In this study, a total of 11,613 circRNAs were expressed in the caprine skin tissue. Among them, 261 circRNAs were differentially expressed in skin tissue between LC goats and ZB goats, with significant differences in cashmere fiber traits. As shown in Figure 6, differentially expressed circRNAs contributed to the regulation of hair follicle development and cashmere fiber traits through the involvement of their parent genes. A circRNA-miRNA network revealed that eight circRNAs play an important role in the regulation of cashmere fiber traits by acting as miRNA sponges. The results provide a solid foundation for further, in-depth investigations into the function of individual circRNA in the cashmere fiber traits of cashmere goats.

## Figures and Tables

**Table 1:** PCR primer used to amplify circRNAs.

CircRNA/Gene	Forward (5′→3′)	Reverse (5′→3′)
circ_009753	TTCTACTGTGATAGGGCTCG	CACATTGAAGTCAGGTGGTT
circ_001316	ACGGGCATGTAGTCACGG	TCAACCCCAGGGCACAGA
circ_003088	GCTCAGGCAAAGATAGAAG	ACTGAAGGTCGAGGGTCT
circ_001762	CAGGTGCCCAGATGACTA	CCTTTAAGCCGTAAGACG
circ_000208	TGCAGGACCTGAGAACAG	TTCACCACCGAGGACAAT
circ_007874	ACACCGTATGGGACAACAA	TCTCAAGGCTCAGCTTCC
circ_000549	TTTAGCCTTGGATTATGAC	TCGCCAGTGTACTTGTTG
circ_006516	TGACCTGGAGCGGCAGAA	GGTGACATAGGACCCAACTGAT
circ_001483	AAACTGCCAATACCCTGAA	AGGTTGCTGCCATGACTT
circ_003977	CTGGAAACTGCCAATACC	CTGCCGTGACTTAGGGAT
*GAPDH*	ACACTGAGGACCAGGTTGTG	GACAAAGTGGTCGTTGAGGG

## Data Availability

The data presented in this study are available on request from the corresponding author.

## Supplementary Materials

The following supporting information can be downloaded at: https://www.mdpi.com/article/10.3390/genes14020331/s1, Supplementary File S1: The differentially expressed circRNAs identified in Liaoning cashmere (LC) goats compared to Ziwuling black (ZB) goats.
